# Spectrophotometric Determination of the Ionization Constant of Dimethylpicric Acid (2,4,6-Trinitro-3,5-xylenol) in Water at 25 °C

**DOI:** 10.6028/jres.064A.054

**Published:** 1960-12-01

**Authors:** Marion Maclean Davis, Maya Paabo, Robert A. Robinson

## Abstract

The ionization constant of dimethylpicric acid (2,4,6-trinitro-3,5-xylenol) in water at 25 °C has been determined by a spectrophotometric method. The *p*K value 1.38 (K ≈0.042) was obtained. Potentiometric titrations, although less precise, yielded a *p*K value of the same magnitude.

## 1. Introduction

Moore and Peck [1]^2^ determined the *p*K of dimethylpicric acid by potentiometric titration, and reported the value 3.3. On the assumption that the effects of substituents on the ionization constant of phenol are additive, data on the ionization constants of phenol and its monosubstitilted derivatives [2, 3] can be used to calculate 1.76 as an approximate *p*K value for dimethylpicric acid. Alternatively, data for 2,6-dinitrophenol [4], *p*-nitrophenol, and 3,5-xylenol [5] can be combined to give another estimate, 1.05. Combining data for 2,6-dinitrophenol and 4-nitro-3,5-xylenol [6] yields the estimate 1.96, whereas utilizing data for 2,4-dinitrophenol [7], *o*-nitrophenol, and 3,5-xylenol leads to the value 1.50. These four estimates, even though not in close agreement, suggest that dimethylpicric acid may be too strong for a reliable application of the method previously used for estimating *p*K [1]. Redetermination of its *p*K by a different method is therefore desirable.

This paper describes the determination of *p*K for dimethylpicric acid by a spectrophotometric procedure.

## 2. Experimental Procedure

### 2.1. Dimethylpicric Acid

Dimethylpicric acid (2,4,6-trinitro-3,5-xylenol) was prepared by nitrating 3,5-xylenol [1]. After isolation by conventional procedures, the product was finally recrystallized from cyclohexane and dried at 90 °C; mp 106.4 to 106.8 °C.^3^ Potentiometric weight-titrations indicated a purity of not less than 99.9 percent.

### 2.2. Determination of *p*K Value by Spectrophotometry

The *p*K value was calculated from the equation

$$pK=-\log\left[H^{+}\right]-2\log\gamma_{\pm }-\log\left[\left(D-D_{1}\right)/\left(D_{2}-D\right)\right],$$ (1)

in which [H^+^] signifies the total hydrogen ion concentration in moles per liter, γ_±_ is taken as the mean activity coefficient of aqueous hydrochloric acid of equivalent molarity,^4^ and the symbols *D*_1_, *D*_2_, and *D* denote the spectral absorbances (optical densities) of solutions containing the same total molar concentration of dimethylpicric acid present as nonionized molecules, ionized molecules, or mixtures of the two, respectively. Dimethylpicric acid is so nearly completely ionized in water at the concentration used (5.004 × 10^−5^ *M*) in determining *D*_2_ and *D*_1_ values that addition of sodium hydroxide (*p*H ≈12) did not measurably alter the absorption curve. In determining *D*_1_ values, the dimethylpicric acid was dissolved in ~2.2*–M* hydrochloric acid. The measurements of *D* values were made with solutions containing 1.250×10^−4^ *M* dimethylpicric acid together with hydrochloric acid in concentrations varying from ~0.01–*M* to ~0.1–*M*. The absorbances were obtained with a Beckman Model DU quartz spectrophotometer.

## 3. Results and Discussion

### 3.1. Spectrophotometric Determination of *p*K

**Figure 1:**
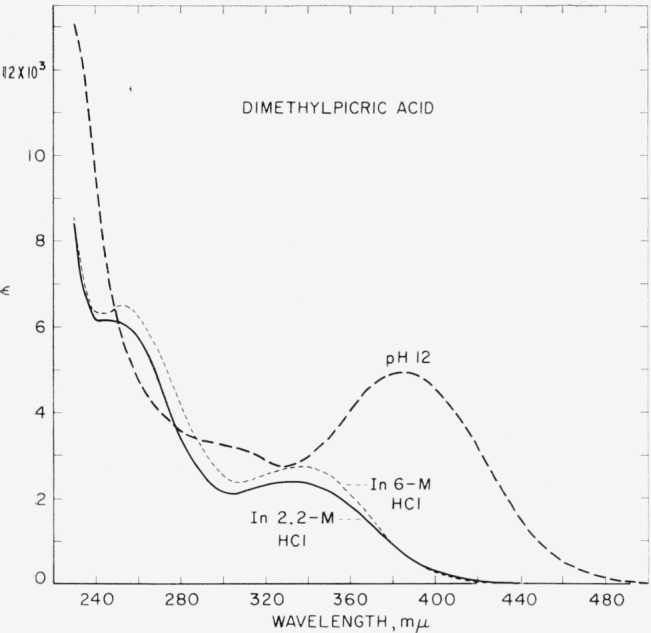
Spectral absorption curves of dimethylpicric acid (2,4,6-trinitro-3,5-xylenol) in aqueous acid (approx. 2.2-*M HCl* and approx. 6–*M HCl*) and in aqueous alkali (*NaOH, pH* ≈12) The curve for the solution containing 2.2*–M* HCl is believed to approximate closely the spectral absorption of nonionized dimethylpicric acid.

Absorption curves for dimethylpicric acid in alkaline and acidic solutions are presented in figure 1. In measuring the absorption of dimethylpicric acid in hydrochloric acid solutions which varied from 1*–M* to 6–*M* in concentration, it was observed that with increasing concentrations of hydrochloric acid the absorption in the region from about 380 m*μ* to about 420 m*μ* gradually decreased to constant values. At wavelengths shorter than 380 m*μ*, however, the absorption at first decreased and then progessively increased. It seems unlikely that this secondary change can be attributed to an impurity in the solutions; possibly, it involves nitro groups. The curves obtained for solutions of dimethylpicric acid in 2.2*–M* and 6*–M* hydrochloric acid illustrate the secondary change. The curve for 2.2–M hydrochloric acid solution is believed to approximate closely the spectral absorption of nonionized dimethylpicric acid.

In determining *p*K, absorbance values at 390, 400, and 410 m*μ* were used. Since the values of *D*_1_ at these wavelengths are low and do not vary measurably with concentrations of hydrochloric acid greater than 3–*M*, the secondary change in absorption is believed not to have caused errors in determining *p*K.

The tabulated data and results are presented in table 1. The average *p*K value obtained was 1.376, or approximately 1.38 (corresponding to an ionization constant of about 0.042). This *p*K value falls within the range of the various estimated constants (see sec. 1).

### 3.2. Attempted Potentiometric Determination of *p*K

Attempts were made to determine *p*K by titrating 0.005–M dimethylpicric acid with alkali about 10 times as concentrated, using glass and saturated calomel electrodes [9].^5^ This concentration of dimethylpicric acid is close to the limit of its solubility in water at 25 °C. Values of *p*K were calculated from the equation

$$pK=pH-\log\{([B^{-}]+[H^{+}])/([HB])-[H^{+}])\}+0.5115\;\surd I/(1+1.5\surd I).$$ (2)

The *p*K values obtained from eight titrations varied from 1.26 to 1.66, yielding the average value 1.49. Considering the limited solubility and relatively high acidity of dimethylpicric acid, high precision and accuracy by potentiometric titration do not appear feasible for this compound. However, the average *p*K value obtained in this work agrees in general magnitude with the value determined spectrophotometrically.

## Figures and Tables

**Table 1:** Ionization constant of dimethylpicric acid (2,4,6-trinitro-3,5-xylenol) in water at 25 °C^a^^,^^b^

Concentration of HCl^b^	*D*	log[(*D-D*_1_)/(*D_2_-D*)]	−log[H+]^b^	−2 logγ_±_	*p*K

λ=390 m*μ* *D*_1_ =0.064, *D*_2_=0.612					

0.0102	0.521	0.701	1.989	0.087	1.375
.0203	.462	.424	1.691	.115	1.382
.0332	.413	.244	1.478	.140	1.374
.0519	.359	.067	1.284	.164	1.381
.0623	.340	.006	1.205	.174	1.373
.104	.277	−.197	0.984	.200	1.381

λ =400 m*μ* *D*_1_=0.029, *D*_2_=0.568					

0.0102	0.477	0.692	1.989	0.087	1.384
.0203	.422	.430	1.691	.115	1.376
.0332	.373	.246	1.478	.140	1.372
.0519	.321	.073	1.284	.164	1.375
.0623	.302	.011	1.205	.174	1.368
.104	.244	−.178	0.984	.200	1.362

λ = 410 m*μ* *D*_1_ = 0.011, *D*_2_=0.495					

0.0102	0.411	0.678	1.989	0.087	1.398
.0203	.366	.440	1.691	.115	1.366
.0332	.318	.239	1.478	.140	1.379
.0519	.271	.065	1.284	.164	1.383
.0623	.254	.003	1.205	.174	1.376
.104	.203	−.182	0.984	.200	1.366

Average					1.376
					K=0.042

a Concentration of dimethylpicric acid, 1.250×10^−4^ *M.* Optical absorption cells were 1 cm in length.

b All concentrations are in moles per liter.

## Footnotes

2 Figures in brackets indicate the literature references at the end of this paper.

3 The mp reported in [1] was 106.5 °C (uncor.).

4 Values of γ_±_ were interpolated from a graph of the mean activity coefficient of hydrochloric acid at 25 ° versus its molality (see [8], table 11-4-1A, p. 547). Harned and Ehler’s function derived for 1:1 electrolytes in water was used to convert molarities of acid to the corresponding molalities (see table 12-1-1A of [8]).

5 In part of the titrations a Precision-Shell titrometer was used; in others, a Beckman *p*H meter, model G.
